# Indian Ornamental Tarantula (*Poecilotheria regalis*) Venom Affects Myoblast Function and Causes Skeletal Muscle Damage

**DOI:** 10.3390/cells12162074

**Published:** 2023-08-15

**Authors:** Nicholas J. Richards, Ali Alqallaf, Robert D. Mitchell, Andrew Parnell, Husain Bin Haidar, José R. Almeida, Jarred Williams, Pradeep Vijayakumar, Adedoyin Balogun, Antonios Matsakas, Steven A. Trim, Ketan Patel, Sakthivel Vaiyapuri

**Affiliations:** 1 School of Biological Sciences, University of Reading, Reading RG6 6UB, UK; n.j.richards@pgr.reading.ac.uk (N.J.R.); a.alqallaf@pgr.reading.ac.uk (A.A.); andrewparnell@micregen.com (A.P.); h.m.binhaidar@pgr.reading.ac.uk (H.B.H.); 2 Medical Services Authority, Ministry of Defence, Kuwait City 13012, Kuwait; 3 Micregen Ltd., Thames Valley Science Park, Reading RG2 9LH, UK; robertmitchell@micregen.com; 4 School of Pharmacy, University of Reading, Reading RG6 6UB, UK; j.r.dealmeida@reading.ac.uk (J.R.A.); j.williams4@pgr.reading.ac.uk (J.W.); pradeep.vijayakumar@pgr.reading.ac.uk (P.V.); 5 Molecular Physiology Laboratory, Centre for Biomedicine, Hull York Medical School, Hull HU6 7RX, UK; 6 Venomtech Ltd., Sandwich CT13 9FE, UK; s.trim@venomtech.co.uk

**Keywords:** *Poecilotheria regalis*, Indian ornamental tarantula, spiders, tarantulas, envenomation, local tissue damage, muscle damage, muscle regeneration, microthrombi, bleeding

## Abstract

Envenomation by the Indian ornamental tarantula (*Poecilotheria regalis*) is medically relevant to humans, both in its native India and worldwide, where they are kept as pets. Muscle-related symptoms such as cramps and pain are commonly reported in humans following envenomation by this species. There is no specific treatment, including antivenom, for its envenomation. Moreover, the scientific knowledge of the impact of this venom on skeletal muscle function is highly limited. Therefore, we carried out this study to better understand the myotoxic properties of *Poecilotheria regalis* venom by determining its effects in cultured myoblasts and in the tibialis anterior muscle in mice. While there was no effect found on undifferentiated myoblasts, the venom affected differentiated multinucleated myotubes resulting in the reduction of fusion and atrophy of myotubes. Similarly, intramuscular administration of this venom in the tibialis anterior muscle in mice resulted in extensive muscle damage on day 5. However, by day 10, the regeneration was evident, and the regeneration process continued until day 20. Nevertheless, some tissue abnormalities including reduced dystrophin expression and microthrombi presence were observed on day 20. Overall, this study demonstrates the ability of this venom to induce significant muscle damage and affect its regeneration in the early stages. These data provide novel mechanistic insights into this venom-induced muscle damage and guide future studies to isolate and characterise individual toxic component(s) that induce muscle damage and their significance in developing better therapeutics.

## 1. Introduction

The Indian ornamental tarantula (also known as Indian ornamental tree spider) (*Poecilotheria regalis*) (hereafter, referred as, *P. regalis*) is native to several regions of India and is recognised as being medically important to humans [1]. Previous studies of envenomations from venomous animals in South India note bites from this species cause inflammation, pain, and swelling [2,3]. In addition to injuries from wild spiders, some case studies report bites from *P. regalis*, which are kept as pets worldwide, including in Europe and the UK [4]. As the species becomes increasingly popular among hobbyists, envenomations are likely to become more common despite advice for safe handling and appropriate housing [4,5]. Symptoms of its envenomation develop over time from hot flushes to severe generalised muscle cramps and aches in the limbs with elevated levels of creatine kinase, which is indicative of muscle damage [1,6]. This venom can induce seizures in mice and an intravenous (IV) lethal dose (LD_50_) of 5–14 mg/kg for the *Poecilotheria* genus was calculated in mice, and specifically, *P. regalis* at approximately 9 mg/kg [7]. There is no antivenom or another specific treatment available for envenomation by this species other than the alleviation of symptoms with anti-inflammatory drugs and benzodiazepines [2]. Therefore, detailed investigations are required to determine the impact of this venom on the modulation of various cellular functions including skeletal muscle and develop better therapeutics to treat the envenomation of this species. Moreover, developing a better understanding of invertebrate venoms will provide a plethora of scientific knowledge and opportunities to develop novel diagnostics and therapeutics for various human diseases using venom toxins as lead compounds [8].

Mammalian skeletal muscle has a remarkable ability to regenerate, a property imbued by the resident stem cells called satellite cells [9]. These mononucleated self-renewing stem cells are found in a specialised niche under the basement membrane surrounding the myofibres of the skeletal muscle. Under normal physiological conditions, these cells are maintained in a quiescent state. However, following muscle damage, satellite cells become activated as marked by the induction of a gene, MyoD, and thereafter, they migrate to the site of injury and proliferate [9]. Following proliferation, these cells differentiate and fuse to form multinucleated myotubes which form muscle fibres [10,11]. Many of the early stages of muscle regeneration can be examined through in vitro experimental approaches using immortalised myoblasts such as the C2C12 mouse myoblast cell line, which is commonly used in muscle research [12,13,14,15,16,17,18]. Moreover, several studies have used in vivo animal models to understand how venoms induce muscle toxicity. For example, intramuscular injection into the tibialis anterior (TA) muscles of rodents (specifically mice) is a commonly utilised in vivo approach to characterise the impact of venoms and their toxins in the modulation of skeletal muscle degeneration and regeneration [19,20]. The TA muscle is specifically targeted, as it is relatively large and amenable to injection. The mouse models are advantageous as they provide the context of the complex systems involved in muscle degeneration and regeneration, a process that requires the orchestrated actions of many cell types in a temporally coordinated manner. Hence, in this study, we utilised both in vitro (C2C12 myoblast cell line) and in vivo (TA muscle injury in mice) models to determine the impact of *P. regalis* venom on key stages of muscle degeneration/regeneration. The results of this study demonstrate that this venom affects the fusion of cultured myoblasts, induces atrophy of myotubes, and significantly damages the TA muscle in mice, although it allows regeneration over time.

## 2. Methods

### 2.1. Cell Culture

The C2C12 myoblast cells were cultured in growth media [Dulbecco’s modified eagle medium (DMEM) with 10% (*v*/*v*) foetal bovine serum (FBS) and 1% (*v*/*v*) penicillin–streptomycin (all from Fisher Scientific, UK)] at 37 °C with 5% CO_2_. Subculturing by passaging was carried out using TrypLE recombinant enzyme (Fisher Scientific, UK) to maintain the cells below 60% confluency. The cells younger than passage 20 were used in all assays in this study.

### 2.2. Cell Viability Assay

C2C12 cells were seeded into 96-well plates at 4000 cells per well in growth media and allowed to adhere for 24 h at 37 °C with 5% CO_2_. *P. regalis* venom (Venomtech Ltd., UK) was diluted in fresh growth media to the desired concentrations (0.1, 1, 10, and 100 µg/mL) and incubated with the cells for 24 h. Images were captured with a 10x objective on an EVOS-XL microscope (ThermoFisher Scientific, UK). Thereafter, MTS [3-(4,5-dimethylthiazol-2-yl)-5-(3-carboxymethoxyphenyl)-2-(4-sulfophenyl)-2H-tetrazolium, inner salt] (317 μg/mL) (Promega, UK) reagent was added and incubated with the cells for one hour, according to the manufacturer’s instructions. Then, the absorbance was measured using a spectrophotometer (Spectramax 340PC 384, Molecular Devices, UK) at 490 nm.

### 2.3. Lactate Dehydrogenase (LDH) Cytotoxicity Assay

A total of 10,000 C2C12 cells were plated per well and allowed to adhere overnight in growth media. The media were then replaced with fresh media containing venom (25, 50, or 100 µg/mL) or the lysis buffer (which acted as a positive control) provided in the kit (CyQUANT^TM^, Invitrogen, UK). Cells included in these conditions were compared to the controls at four or 24 h. Thereafter, 25 µL of media or supernatant was mixed with 25 µL of the LDH substrate solution for 30 min at 37 °C. Following the incubation, the reaction was terminated by the addition of 25 µL of the stopping reagent, and the absorbance was measured at 490 nm with a spectrophotometer.

### 2.4. Cell Migration Assay

Time-lapse microscopy was used to image the cells plated at a low density (3000 cells per well) in a 24-well plate and incubated at 37 °C with 5% CO_2_ for 24 h to permit cell adherence. Thereafter, the cells were incubated with various concentrations of venom, and images were taken at 10 min intervals for 24 h using a brightfield TIE microscope (Nikon Ti-E, UK) with a 10× objective. The migration speed was calculated using the MtrackJ application on ImageJ software (version 1.53) (NIH, USA).

### 2.5. Scratch Assay

C2C12 cells were grown in a 24-well plate until a confluency of 80% was reached. Then, venom at different concentrations (1 and 10 µg/mL) was added to new growth media and added to the cells, and they were allowed to grow for six hours. Following incubation, a scratch was introduced with a pipette tip at the middle of each well. Images of the cells around the scratched area were obtained using timelapse microscopy, at 10 min intervals over a period of 14 h. The timepoint at which the first cell from one side contacted a cell from the other side was taken as the time to closure or the migration time.

### 2.6. Fusion Assay

C2C12 cells were seeded at a density of 50,000 cells per well onto 16 mm pre-prepared acid-etched coverslips in 12-well plates. The cells were then incubated in 1 mL of growth media at 37 °C and 5% CO_2_ until 95% confluency was reached (~48 h). The cells were then incubated for 5 days in differentiation media [high glucose DMEM with 5% (*v*/*v*) horse serum] at 37 °C with 5% CO_2_. For the fusion assay, the differentiation media contained no venom (negative control) or various concentrations of venom (0.1, 1, and 10 µg/mL). After 5 days of incubation, the cells were fixed for 20 min in 2% (*w*/*v*) paraformaldehyde in PBS 1:1 in the differentiation media and stored in PBS at 4 °C before immunocytochemistry.

### 2.7. Atrophy Assay

C2C12 cells were seeded at a density of 50,000 cells per well on 16 mm acid-etched coverslips in 12-well plates and differentiated to form myotubes without venom for 5 days at 37 °C with 5% CO_2_. The differentiation media were then replaced with fresh differentiation media containing venom (0.1, 1, and 10 µg/mL) and incubated for a further 24 h. The myotubes were then fixed for 20 min in 2% (*w*/*v*) paraformaldehyde in PBS 1:1 in the differentiation media and stored in PBS. The area of myotubes was determined by measuring the length and width of each myotube using ImageJ. The widths were measured at different regions of each myotube with at least four measurements in each but more in the longer myotubes. The measurements for widths were then averaged and multiplied by the length to calculate the myotube area.

### 2.8. Immunocytochemistry

Cells on coverslips were washed three times with PBS (5 min each) before permeabilisation buffer [20 mM HEPES pH 7, 300 mM sucrose, 50 mM NaCl, 3 mM MgCl_2_, and 0.5% (*v*/*v*) Triton X-100] was applied at room temperature for 15 min, followed by a second round of washes (three times) with PBS. Blocking of nonspecific binding was achieved using a blocking wash buffer [5% (*v*/*v*) horse serum, 0.05% (*v*/*v*) Triton X-100, and 25 mg sodium azide in PBS] for 30 min. Immunostaining for MYH1 was performed using the primary antibody, anti-MYH1 mouse IgG (1:1 supernatant, DHSB Clone: A4.1025, prepared in-house), which was incubated with the myotubes overnight at 4 °C. The myotubes were washed in a blocking wash buffer three times, for 10 min each. The secondary antibody, Alexa Fluor^TM^ 594 fluorophore-conjugated goat anti-mouse IgG (1:200, Invitrogen, UK), was diluted in blocking wash buffer, and the myotubes were incubated with the secondary antibodies for one hour in the dark before three 10 min washes using blocking wash buffer. The coverslips were then mounted onto slides, and the DNA stain 4′,6-diamidino-2-phenylindole (DAPI) (2.5 μg/mL) was added into the mounting medium (Dako, UK). Images were captured using an epifluorescence microscope with a 5x objective (Zeiss AxioImager, UK). Composite images were created in Adobe Photoshop (Version CS6) (Adobe, USA). Measurements of the length and width of the myotubes were used to determine the area in the atrophy assay, while the nuclei visualised using DAPI were counted to establish the fusion index for the fusion assay defined as the number of nuclei inside myotubes divided by the total number of nuclei in a field of view multiplied by 100 to derive the percentage.

### 2.9. Enzymatic Assays

The enzymatic assays for venom were performed as described previously [19,21,22,23]. A dye quenched (DQ) gelatin (ThermoFisher Scientific, UK) (50 μg/mL) was used as a fluorescent substrate for metalloproteases. The substrate was incubated with the venom at 50 μg/mL in 100 μL total reaction volumes at 37 °C, and fluorescence readings were taken every 10 min for 90 min with a spectrofluorometer (FLUOstar OPTIMA, BMG Labtech, Germany) with an excitation wavelength of 485 nm and emission at 520 nm. Similarly, Nα-Benzoyl-L-Arginine-7-Amido-4-methylcoumarin hydrochloride (BAAMC) (2 μM) was used as a substrate to measure venom serine protease activity using an excitation wavelength of 366 nm and emission at 460 nm. The venom of the Western Diamondback Rattlesnake (*Crotalus atrox*), a viper species known to have high levels of metalloprotease and serine protease activities [24] was used at 50 μg/mL as a positive control. The PLA_2_ activity was measured using the EnzChek^TM^ phospholipase A_2_ (PLA_2_) assay kit (ThermoFisher Scientific, UK) in line with the manufacturer’s protocols. To assess nonspecific protease activity, chromogenic azocasein (Merck, UK) was used as a substrate at 5 mg/mL in 50 mM Tris-HCl (pH 8.0) buffer [25]. The venom concentrations (25, 50, and 100 μg/mL) were diluted in 90 μL of the substrate solution. The venom of *Crotalus atrox* was used as a positive control. After incubating for 90 min at 37 °C, 200 μL of 5% trichloroacetic acid (TCA) was added to each reaction and centrifuged for 5 min at 8000 rpm. The supernatant (150 μL) was placed in a 96-well plate, to which 150 μL of NaOH (0.5 M) was added. The formation of TCA-soluble azopeptides was measured at 440 nm using a spectrofluorometer.

### 2.10. SDS-PAGE and Silver Staining

The venom (10 µg) was prepared in a 6× reducing sample treatment buffer [12% (*w*/*v*) SDS, 30% (*v*/*v*) β-mercaptoethanol, and 30% (*v*/*v*) glycerol in 0.5M Tris-HCl (pH 6.8), with traces of bromophenol blue). The venom was denatured by heating at 95 °C for 10 min. Molecular weight standards (Novex sharp pre-stained protein standard, Invitrogen, UK) and venom proteins were resolved using a pre-cast gel (4–15%) Tris-glycine gel (Bio-Rad, UK). The proteins were visualised using the Silver Xpress kit (Invitrogen, UK).

### 2.11. Venom-Induced Muscle Damage in Mice

The British Home Office (project license number: PP8746932) and University Research Ethics Committee approved all the procedures performed on mice in this study. C57BL/6 male (because of their larger muscle size and lower variability, which make the analysis robust by obtaining consistent results) mice (12–13 weeks old) were anaesthetised with 3.5% (*v*/*v*) isoflurane in O_2_ and maintained at 2% (*v*/*v*) isoflurane during injections. *P. regalis* venom (1 µg/g of mouse weight) in 30 μL of phosphate-buffered saline (PBS) was injected intramuscularly into the left TA muscle, and then the mice were allowed to recover. At each time point of 5-, 10-, and 20-days post-injection, five mice were sacrificed. The TA muscles injected with the venom and the contralateral TA of each animal were dissected. These muscles were weighed before freezing on liquid-nitrogen-cooled isopentane before storage on dry ice and then at −80 °C. Venom-injected TA muscle weights were expressed as a percentage weight compared to the contralateral (non-injected) control muscle weights, which were then averaged for each cohort. Data of the normalised muscle mass were then developed in a graph showing the control value set to 100%. Frozen TA muscles were blocked in OCT (Optimal Cutting Temperature) compound (CellPath Ltd., UK). Transverse 15 µm sections were collected using a cryostat (Bright Instruments, UK) and then transferred onto microscope slides in serial sections before storage at −80 °C.

### 2.12. Histological Staining

After warming slides at room temperature for 15 min and two minutes of washing with PBS to remove the OCT, Harris haematoxylin (Sigma Aldrich, UK) was used for two minutes to stain the nuclei. Then, the slides were washed with double distilled water for two minutes prior to the differentiation of the stain, using two rapid immersions and egressions in 70% ethanol with 0.5% hydrochloric acid. Running tap water was then used as a wash for 5 min. Then, 1% (*v*/*v*) Eosin (Sigma Aldrich, UK) (in 70% ethanol) was used as a counterstain. Dehydration was then performed using an ethanol series followed by xylene. Finally, a glass coverslip was fixed using a DPX mounting medium. The slides were imaged using a Hamamatsu Photonics NanoZoomer-SQ digital slide scanner (Japan) and processed using the NanoZoomer digital pathology software, Adobe Photoshop (Version CS6) (Adobe, USA) and ImageJ (USA). For picrosirius red staining, TA muscle sections on slides were immersed into Bouin solution [5% (*v*/*v*) acetic acid, 9% (*v*/*v*) formaldehyde, and 0.9% (*v*/*v*) Picric acid] for 15 min in a 56 °C water bath to stain the cytoplasm. Following this, the slides were washed with running tap water for 15 min. After tapping dry, the slides were stained in picrosirius red stain (Abcam, UK) for an hour at room temperature in the dark. The muscle sections were twice dipped in two changes of acidified water [0.5 (*v*/*v*) glacial acidic acid in water]. Three 5 min washes were performed using 100% ethanol followed by 5 min in xylene. Finally, DPX mounting media was used to fix the coverslips in place before being left to dry overnight. The sections were then imaged under brightfield light microscopy at 10× magnification.

### 2.13. Immunohistochemistry of TA Muscle

Immunohistochemistry was performed as reported by us previously [19], similar to the sections with fixed myotubes. After warming at room temperature, the sections were washed with PBS to remove the OCT. The permeabilisation buffer (as above) was then applied for 15 min before three washes with PBS followed by incubation with a blocking washing buffer for 30 min. Primary antibodies were blocked in the blocking-wash buffer for 30 min before incubation on the muscle sections at 4 °C overnight. The primary antibodies used in this study were mouse anti-Myosin heavy chain 3, 1:200, (Santa Cruz, SC53091, USA), Rabbit anti-Dystrophin, 1:200, (Abcam, 15277, UK), Rabbit anti-Laminin, 1:200, (Sigma Aldrich, L9393, UK), and F4/80 marker of macrophages 1:100 (Santa Cruz, SC25830, UK). The slides underwent three 5 min washes to remove the unbound primary antibodies before the secondary antibodies conjugated with different Alexa Fluor^TM^ fluorophores, (blocked using blocking-wash buffer for a minimum of 30 min prior to use) were applied for one hour in the dark. The secondary antibodies were goat anti-mouse or anti-rabbit IgG (H+L), 1:200, either Alexa Fluor 488 or 594, (all Invitrogen, A11029, A11032, A11034, A11037, UK). Further, a final washing of the unbound antibodies was performed prior to mounting and imaging them. Dystrophin perimeter circularity was measured using the perimeter of each fibre within or partially within 200 µm^2^ fields of view. Expression around the full perimeter was 100%. For fibres not expressing dystrophin at 100% as indicated by disruption to the expression domain, the perimeter that did express dystrophin was measured and presented as a percentage of the whole perimeter of the fibre. Areas where the fibre structure had been lost and had zero percentage of dystrophin expression were not considered in this analysis. Laminin intensity was measured using ImageJ. Lines were drawn at several points across the thickness of the laminin domain surrounding each of the fibres within 200 µm^2^ fields of view. The maximum grey value intensity of the lines for each 200 µm^2^ field of view was averaged. Fibrinogen was analysed using threshold analysis applied to whole muscle sections. To visualise the acetylcholine receptors localised to motor neuron endplates as a maker of neuromuscular junctions in the cross-sectioned muscle, α-bungarotoxin from the venom of the snake *Bungarus multicinctus* conjugated to Alexa Fluor^TM^ 647 (Invitrogen, UK Cat# B35450) was used (1:1000) following washing and blocking as above. Neuromuscular junctions in TA muscle were measured for changes in length compared to the contralateral controls in ImageJ (USA). For all immunohistochemistry analyses, fluorescent mounting medium containing DAPI to visualise nuclei was used to affix coverslips to slides, before imaging via epifluorescent microscope (Zeiss AxioImager, UK) and ImageJ analysis.

In summary, for quantification of the expression domains, the following areas were used: picrosirius red and fibrinogen 600 µm^2^ and 200 µm^2^ for IgG, MHC3, dystrophin, laminin and P-selectin, and entire muscle for BTx (due to its low abundance). These areas were selected based on the analysis package used and the ease of quantification to gather meaningful data for the analysis.

### 2.14. Statistical Analysis

Statistical analyses were performed using GraphPad Prizm 5, and *p*-values were calculated using one-way ANOVA with Bonferroni’s multiple comparison test to compare the control with all other conditions and all conditions with each other. Unpaired two-tailed T-tests were also used when necessary. Data are shown as the mean with error bars depicting the standard error of the mean (SEM).

## 3. Results

### 3.1. Biochemical Characterisation of P. regalis Venom

First, we investigated the presence of key muscle-damaging enzymes such as PLA_2_ and metalloproteases as well as serine proteases (that are often associated with blood clotting and thereby causing ischaemia) in this venom using selective fluorescence-based enzymatic assays. There was no significant metalloprotease (Supplementary Figure S1A), serine protease (Figure S1B), or PLA_2_ (Figure S1C) activity found in this venom. Moreover, a caseinolytic assay was performed as a measure for diverse proteolytic enzymes using casein as a real substrate, but this venom did not exhibit any caseinolytic activity (Figure S1D). The absence of PLA_2_ and caseinolytic activity in this venom was also previously reported [26]. To analyse the protein content of this venom, we used gel electrophoresis followed by silver staining. This revealed that this venom contains many proteins with molecular weights ranging from around 110 to less than 3.5 kDa (Figure S1E). These data demonstrate that this venom contains a range of different proteins but lacks key muscle-damaging enzymes.

### 3.2. P. regalis Venom Does Not Affect the Survival and Migration of Myoblasts

**Figure 1:**
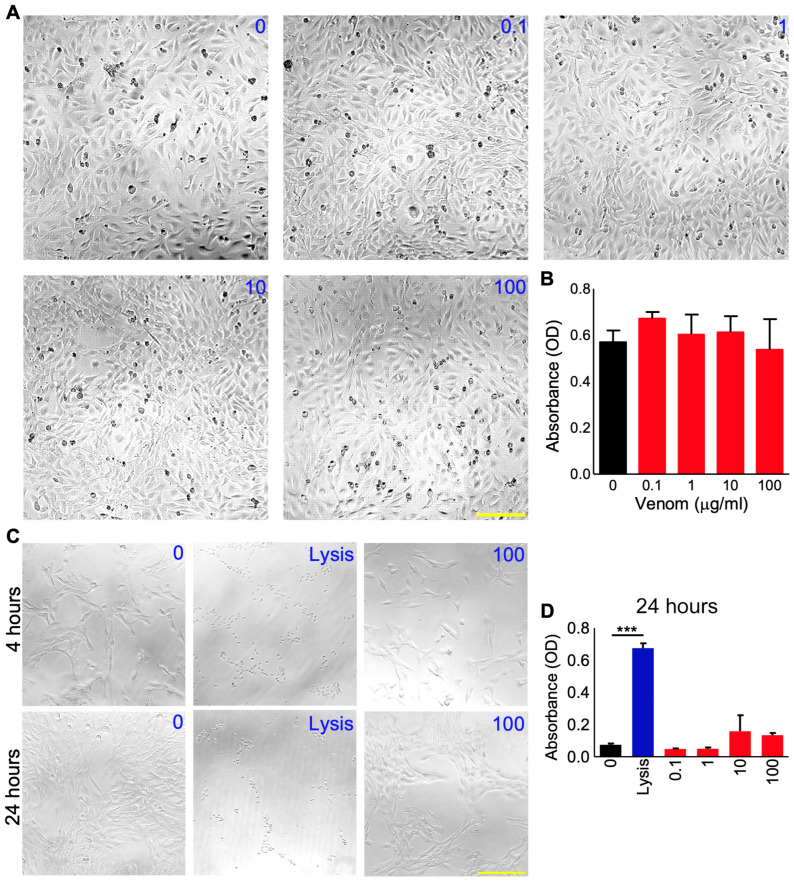
Effects of *P. regalis* venom on the survival of C2C12 myoblast cells. The morphology (**A**) and viability (assessed using an MTS reagent) (**B**) of C2C12 cells were analysed after 24 h of incubation with various concentrations (0 to 100 µg/mL) of venom. Similarly, (**C**) the effects of a range of concentrations of venom on C2C12 cells were assessed using a lactate dehydrogenase assay at four and 24 h following treatment. A lysis buffer containing Triton-X 100 (Lysis) was used as a positive control in this assay. The images shown are representative of three independent experiments, and the quantified data (**D**) show the level of absorbance obtained in this assay. Data represent the mean ± SEM (*n* = 3). The scale bars represent 200 µm and are applicable to all the images in each panel. The *p*-value shown (*** *p* < 0.001) was calculated using one-way ANOVA.

To determine the effects of *P. regalis* venom on the early stages of muscle development, the survival and migration of C2C12 mouse myoblasts were analysed in the presence and absence of this venom. A range of different concentrations (0.1 to 100 μg/mL) of venom was incubated with cultured C2C12 cells for 24 h, and then a cell viability assay was performed using the MTS reagent. The microscopic examination of the cells following treatment with venom shows that the cells were not significantly affected by the venom, as there were no morphological abnormalities (Figure 1A). The results from the assay confirm that the venom did not affect the viability of the C2C12 cells at all the concentrations tested (Figure 1B). These results were corroborated using the LDH cytotoxicity assay, which displayed no toxicity of this venom both at 4 and 24 h (Figure 1C,D). These data suggest that this venom does not affect the viability and proliferation of C2C12 cells through cytotoxic effects.

**Figure 2:**
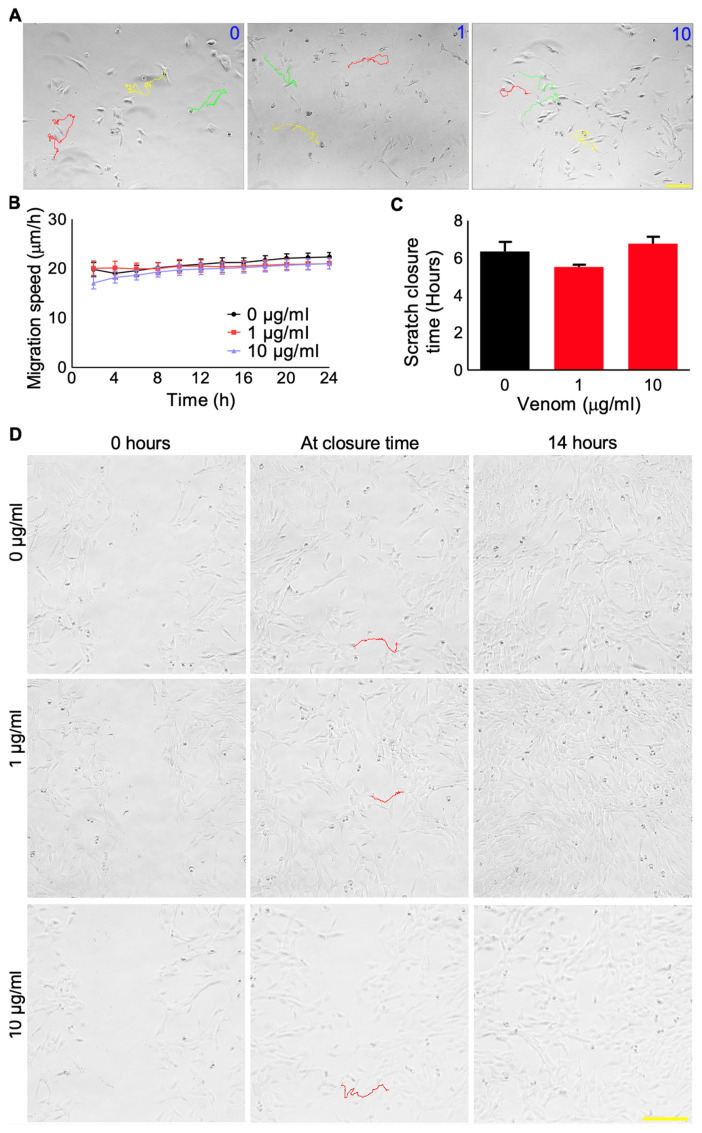
Effects of *P. regalis* venom on the migration of C2C12 cells. (**A**) The migration tracks (coloured lines) of individual C2C12 cells without and with different concentrations of venom (0, 1, and 10 µg/mL) were analysed for 24 h. (**B**) The quantification of the migration speed of individual cells over 24 h. Data represent the mean ± SEM. (*n* = 18). In addition, a scratch assay (**C**,**D**) was performed to determine the mass cell migration upon treatment with different concentrations of venom (0, 1, and 10 µg/mL). The time taken for the first cell from one side to meet with another cell from the other side was taken as the scratch closure time. The images were also captured at 14 h when the assay was terminated as the complete closure of the scratch was achieved. Data represent the mean ± SEM (*n* = 8).

The individual migration of muscle stem cells is critical during the formation and regeneration of skeletal muscle [9]. Hence, the impact of this venom on the migration of C2C12 cells was examined using time-lapse microscopy. This venom did not significantly affect the migration of C2C12 cells at any of the concentrations tested (Figure 2A,B). Moreover, the migration of cells was also examined using a scratch assay, which further confirmed that this venom does not have an impact on cell migration (Figure 2C,D). These data suggest that this venom does not affect the early stages of muscle development in the C2C12 cells.

### 3.3. P. regalis Venom Affects the Fusion of Myoblasts into Myotubes and Induces Atrophy

**Figure 3:**
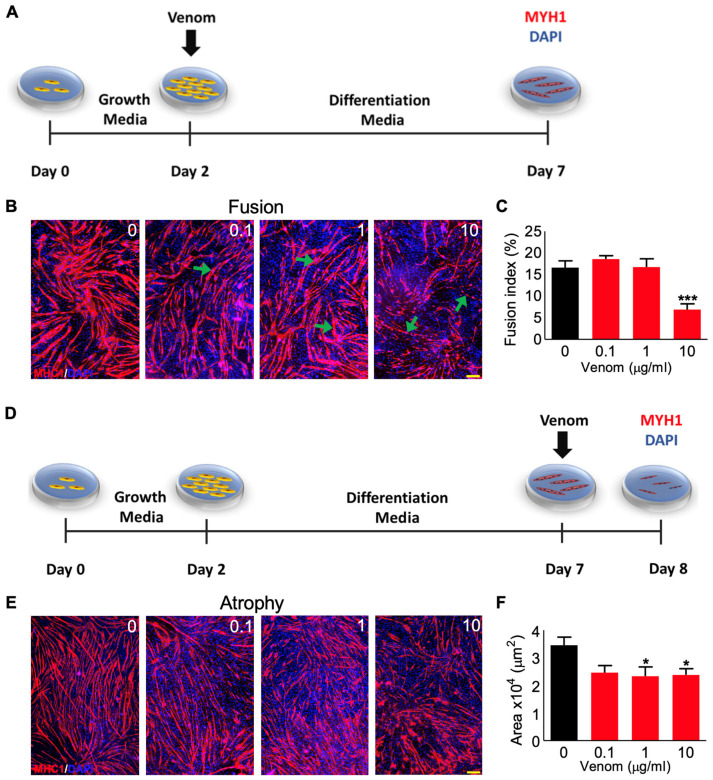
Impact of *P. regalis* venom on the fusion of myoblasts into myotubes and atrophy of myotubes. (**A**) Schematic representation of the protocol used for fusion assay. (**B**) Staining of myotubes for MHC1 on day 7 (affected areas are indicated by arrows). (**C**) Quantification of the fusion index of the myotubes (*n* = 11). (**D**) Schematic representation of the protocol used for the atrophy assay. (**E**) Staining of myotubes for MHC1 on day 8. (**F**) Quantification of the myotube area (*n* = 20). Data represent the mean ± SEM. *p* values shown were calculated by one-way ANOVA followed by the Bonferroni post-test (* *p* < 0.05, and *** *p* < 0.001). The scale bars represent 200 µm, and they are applicable for all images in each panel.

The fusion of mononucleated myoblasts into multinucleated myotubes is required for successful muscle development and regeneration [10]. Therefore, the effects of *P. regalis* venom in the fusion of C2C12 cultured cells into myotubes were analysed. The cells were cultured at high density for 2 days using the normal growth media, and then they were kept in a low growth factor differentiation media to facilitate them to fuse into myotubes for another 5 days in the presence and absence of different concentrations of venom (Figure 3A). The newly formed multinucleated myotubes expressed myosin heavy chain 1 (MHC1) (Figure 3B). The fusion index was calculated as the number of nuclei inside myotubes divided by the total nuclei in a field of view multiplied by 100 to derive the percentage for the fusion assay. The results demonstrate that the formation of myotubes (Figure 3B) and fusion index (Figure 3C) were not affected by low concentrations (0.1 and 1 µg/mL) of venom, although they were significantly reduced at 10 µg/mL of venom. Similarly, the ability of the venom to induce atrophy was examined by exposing differentiated myotubes (after 7 days) to different concentrations of venom for 24 h (Figure 3D) and then assessing their impact on the size of myotubes. There was no significant impact at the lowest concentration of venom (0.1 µg/mL); however, significant atrophy was observed at concentrations of 1 and 10 µg/mL of venom (Figure 3E,F). These data suggest that *P. regalis* venom mainly affects the later stages (fusion of myoblasts and atrophy of myotubes) of muscle development, and myotubes are more sensitive to this venom compared to myoblasts.

### 3.4. Administration of P. regalis Venom in TA Muscle Induces Muscle Loss in Mice

**Figure 4:**
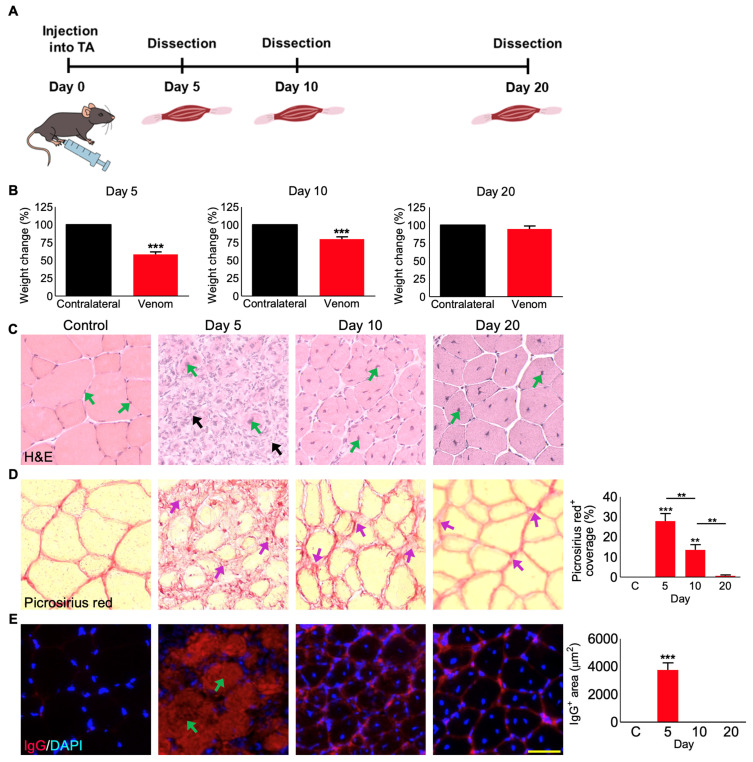
*P. regalis* venom induces weight loss and damage in the tibialis anterior muscle in mice. (**A**) Graphical representation of the protocol used for the in vivo muscle damage experiment in mice. (**B**) The percentage of weight change for the venom-injected TA muscles of C57BL/6 mice compared to their contralateral TA muscles at day 5, 10, and 20 following venom administration. The muscle weights were measured in milligrams, and they were normalised by taking contralateral control muscles as 100%. (**C**) H and E staining of the muscle sections of the undamaged contralateral control and the venom-injected muscles at different time points [green arrows indicate the peripheral nuclei in the undamaged contralateral muscle and the centrally located nuclei (CLN) in the regenerating fibres on days 5, 10, and 20. The black arrows on day 5 indicate the infiltration of immune cells]. (**D**) Picrosirius red staining for fibrosis (indicated by purple arrows) and quantification of the percentage of staining compared to the contralateral controls. (**E**) Intra-fibre IgG localisation (arrows indicate the infiltration of IgG) and quantification of the area of infiltrated necrotic fibres that were fully or partially affected in selected 200 µm^2^ areas. Data represent the mean ± SEM (*n* = 5 mice in each cohort). *p* values (** *p* < 0.01 and *** *p* < 0.001) shown were calculated by one-way ANOVA followed by the Bonferroni post-test except for (**B**) in which student’s *t*-test was used. The scale bar represents 50 µm and is applicable to all images.

Following the assessment of *P. regalis* venom on cultured mouse myoblasts and myotubes, the impact of this venom was studied in mice under in vivo settings. The venom [25 µg in 30 µL of PBS for a 25 g mouse (i.e., 1 µg/g, 1 mg/kg)] was administered intramuscularly (IM) into the left-side TA muscle of anaesthetised C57BL/6 male (because of the larger muscle and its robustness for analysis) mice. Following recovery from anaesthetics, the venom injection did not result in any long-term lameness or compromised mobility, and the overall mouse weights did not significantly decrease. No overt signs of disturbances to the nervous system were apparent (e.g., convolutions), and the mice were always alert and inquisitive following the procedure, exhibiting little to no visible signs of stress (lack of movement or interest in the environment nor any facial grimacing). These findings relate to the low concentration (1 mg/kg) of venom that we administered locally (IM) in muscle in this study compared to the reported IV LD_50_ value of 9 mg/kg. The mice were then sacrificed at different time points (5, 10, and 20 days) (Figure 4A) to analyse the effects. The weights of the venom-treated muscles on days 5 and 10 were significantly reduced compared to the control (contralateral) muscles, although there was no difference observed on day 20 (Figure 4B). These data illustrate that *P. regalis* venom induces a loss in muscle mass of 42% within 5 days following its administration, but the muscle mass recovered gradually to a 21% loss at 10 days, and at 20 days, there was no significant difference to the control weights. 

### 3.5. P. regalis Venom Induces Fibrosis and Infiltration of IgG in TA Muscle at Early Time Points

A detailed histological examination of the venom-damaged TA muscle was performed to understand the mechanisms that drive this damage. Haematoxylin and Eosin (H&E) staining of muscle sections revealed the structural changes in the muscle fibres following treatment with the venom (Figure 4C). At day 5, high levels of tissue damage together with cellular infiltration and a loss of the organised myofibre structure were evident in the venom-treated muscle compared to the undamaged regions of the same or the control muscle. By day 10, the presence of structured fibres with centrally located nuclei (CLN) was clearly visible, indicating that muscle regeneration had occurred. Notably, regeneration was more evident on day 20 with CLN. In contrast to healthy/undamaged muscle fibres that possess nuclei at the periphery (Figure 4C), the regenerating/regenerated fibres will only have CLN, which may not move to the periphery for a long time. The level of fibrosis in muscle sections was quantified using a picrosirius red stain (Figure 4D). At day 5, the stained area accounted for nearly 25% of the total muscle compared to the controls. Fibrosis was still evident at day 10, although at reduced levels compared to day 5. The fibrosis was completely reduced by day 20 compared to day 5 and 10. The integrity of the myofibre was assessed by quantifying the infiltration of the circulating IgG (which are normally present in the blood but enter damaged tissues following injury) into muscle fibre (Figure 4E). This analysis revealed high levels of compromised muscle fibres at day 5 as demonstrated through the excessive infiltration of IgG, but the IgG could not be detected at muscle sections obtained at later time points. In addition, macrophages were located using F4/80 as a marker (Supplementary Figure S2). The number of macrophages increased on day 5 but reduced by day 10. These results demonstrate that *P. regalis* venom causes significant damage to skeletal muscle in mice, but the muscle retains its ability to regenerate over time, for example, by day 20.

### 3.6. Muscle Regeneration following P. regalis Venom-Induced Damage

**Figure 5:**
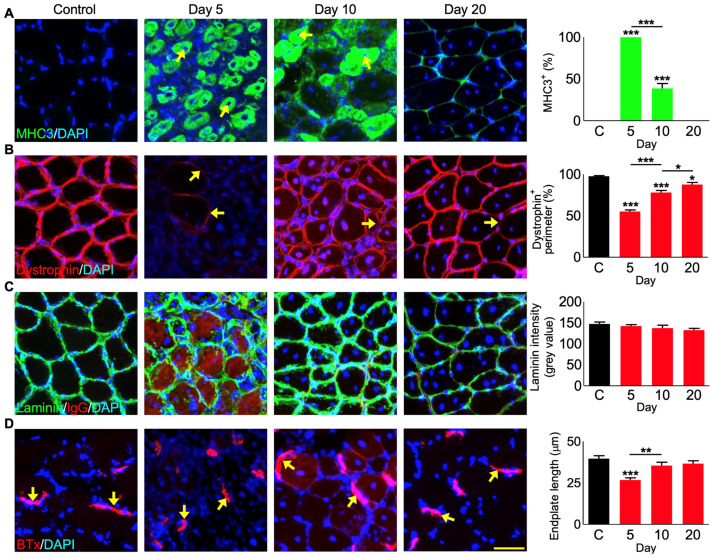
Regeneration of the tibialis anterior muscle following *P. regalis* venom treatment. (**A**) Expression of myosin heavy chain 3 (MHC3) (arrows showing MHC3 expressing fibres) and quantification of areas expressing MHC3. (*n* = 5 mice for each cohort). Similarly, (**B**) expression of dystrophin (arrows showing low expression areas) and quantification of dystrophin perimeter values (*n* = 48 images for each cohort. All images were obtained from five mice in each cohort). (**C**) Expression and quantification of the intensity of fluorescence for laminin (*n* = 5 mice for each cohort). (**D**) α-bungarotoxin localisation and measure of the size of the neuromuscular junctions (*n* = 36 junctions measured per group, each group had 3 mouse muscles). Data represent the mean ± SEM. *p* values (* *p* < 0.05, ** *p* < 0.01 and *** *p* < 0.001) were calculated by one-way ANOVA followed by Bonferroni post-test. The scale bar represents 50 µm and is applicable to all images. ‘C’ represents contralateral muscles that were collected on day 10.

Expression of myosin heavy chain three (MHC3) is a marker for muscle fibre regeneration [27,28]. Therefore, this marker was analysed in the venom-damaged muscle sections by immunohistochemistry. While the control muscle did not express MHC3 as expected, it was abundantly expressed in almost 100% of the fibres in the damaged areas on day 5 (Figure 5A). However, on day 10, only around 40% of the fibres expressed MHC3, and by day 20, the regeneration had progressed further with no expression of MHC3 detected. Moreover, dystrophin is used as a marker to inform muscle function based on its ability to form the core around which the dystrophin-associated protein complex forms, which is critical for membrane integrity [29]. It should normally be expressed uniformly around the myofibre. Therefore, establishing the expression of dystrophin can be used as a proxy for mature fibres. We measured the expression of dystrophin as a percentage of each fibre’s perimeter, known as circularity, and found a clear reduction in the dystrophin circularity in the damaged areas of the muscle sections on day 5 (Figure 5B). It is important to note that there were many fibres that had robust MHC3 expression but no expression of dystrophin to measure at day 5. On days 10 and 20, incremental increases in percentage expression were shown from a low 55% dystrophin expression per fibre in the damaged areas on day 5 (discounting fibres without any dystrophin measurable) to 78% on day 10 and 88% on day 20. Importantly, circularity at day 20 remained significantly decreased compared to the controls. The intensity of laminin was measured as a marker for the extracellular matrix (ECM) [30]. The levels of laminin were unchanged in the venom-treated muscles compared to the controls at any muscle sections collected at different time points (Figure 5C). This indicates that *P. regalis* venom does not affect the ECM, and the intact ECM may support the organisation and regeneration of muscle fibres.

### 3.7. P. regalis Venom Affects Neuromuscular Junctions

The neuromuscular junctions between the nervous system and muscle are an important indication of the return of functionality for newly regenerated fibres [31]. The motor neuron endplates were visualised using α-bungarotoxin conjugated with Alexa Fluor^TM^ 647 [32,33]. The α-bungarotoxin binding domains to acetylcholine receptors were measured to report on the maturity of the neuromuscular junctions by comparing the motor neuron endplates with the controls. These junctions were found to be decreased in length at day 5 compared to the controls (an average of 26.7 µm on day 5 compared to 39.5 µm for the controls) (Figure 5D). However, they attained lengths similar to the controls by day 10 and 20 (35.4 and 36.6 µm, respectively).

### 3.8. P. regalis Venom Induces Bleeding and Microthrombi Formation in Damaged Muscle

**Figure 6:**
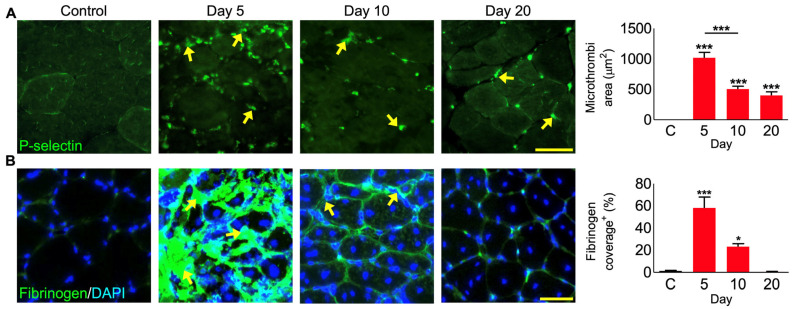
Impact of *P. regalis* venom in inducing bleeding and blood clots in the tibialis anterior muscle in mice. (**A**) Localisation of the P-selectin exposure on the surface of platelets in muscle sections. The graph shows the quantification of the total microthrombi area per 200 µm^2^ view field (*n* = 24 images and 5 mice for each cohort). (**B**) Localisation of fibrinogen in muscle sections and the graph shows the quantification of percentage fibrinogen coverage per damaged area with representative images showing areas of 200 µm^2^ (*n* = 5 mice for each cohort). Data represent the mean ± SEM. *p* values (* *p* < 0.05 and *** *p* < 0.001) were calculated by one-way ANOVA followed by the Bonferroni post-test. The arrows indicate the areas with microthrombi in muscle sections. The scale bars represent 50 µm. ‘C’ represents the contralateral control muscles that were collected on day 10.

As blood circulation is critical for muscle survival, function, and regeneration [34], the impact of the venom on bleeding and microthrombi formation was analysed in venom-damaged muscle sections using specific biomarkers. P-selectin exposure on the surface of platelets as a marker for α-granule secretion indicates the activation of platelets and their aggregation into microthrombi in the capillaries supporting muscle function. Microthrombi were totally absent in the control muscle, but they were clearly present in the venom-damaged muscle on days 5, 10, and 20 (Figure 6A). The levels of microthrombi were largely reduced on days 10 and 20 compared to day 5. Fibrinogen was used as another marker to demonstrate the level of bleeding as well as microthrombi. Compared to the control muscles which did not show any fibrinogen, the level of fibrinogen was increased at day 5 with an average area of around 60% in each section being covered (Figure 6B). A decrease in the fibrinogen level was observed on day 10 and it was absent by day 20. These data indicate that *P. regalis* venom disrupts haemostasis in and around damaged tissues in addition to impacting the muscle fibres.

## 4. Discussion

A better understanding of invertebrate venoms, their composition, and their mode of action would be highly beneficial not only to understand their envenomation pathology in humans but also to reveal their potential for biomedical, biotechnological, and clinical applications. This is exemplified by discoveries including the Hi1a, a disulphide-rich peptide from the Australian funnel-web spider, *Hadronyche infensa,* which exhibited neuroprotective effects in patients following a stroke [35]. Selective spider venom peptides also show significant antimicrobial properties [36,37], which might be useful to tackle the increasing microbial resistance to currently used antibiotics. For example, the biological screening of selective spider and scorpion venoms has revealed valuable molecular scaffolds including the antimicrobial peptides La47 and Css54, which constitute promising candidates for the innovative treatment of infections caused by drug-resistant bacteria [38]. Similarly, proteomics and the functional characterisation of selective invertebrate venoms have shed light on the anticancer effects of their toxins, which has led to the development of potential anticancer agents such as chlorotoxin (from the venom of the Giant Yellow Israeli scorpion, *Leiurus quinquestriatus*) and instigated a clinical trial for its synthetic analogue against glioma [39]. In this study, we investigated the impact of *P. regalis* venom in the modulation of myoblast function and inducing skeletal muscle damage in mice. This tarantula is native to India but is kept as a pet in many parts of the world. The bites from this species have been reported previously, and they are known to induce muscle cramps [7]. Therefore, the effects of *P. regalis* venom were analysed in myoblasts and skeletal muscle in mice to determine their specific actions on skeletal muscle and explore its therapeutic values.

Metalloproteases and phospholipases in the venoms of snakes specifically vipers and spiders are the key modulatory enzymes for causing muscle damage [19,40,41,42,43,44,45,46]. Our initial analysis confirmed that *P. regalis* venom does not contain metalloproteases, serine proteases, non-specific proteases, or PLA_2_ activities. These findings of fluorescence- and absorbance-based detection coincide with an earlier study of this spider venom, which reported a lack of caseinolytic and PLA_2_ activity using absorbance-based substrates [26]. However, the venom was found to have high hyaluronidase activity in this previous study [26]. Spider venoms are complex biological mixtures including enzymes and non-enzymatic peptides, and some of these peptides were shown to exhibit myotoxic actions [42,43]. For example, partitagin, a metalloprotease from a spider (*Hippasa partita*) venom exhibited tissue necrosis activity [42]. Covalitoxin-I is a short peptide characterised by the venom of *Coremiocnemis validus* (Singapore tarantula) to induce myonecrosis in a mouse model [47]. Another relevant example is a short basic peptide of 6.7 kDa from the tarantula *Dugesiella hentzi* (Girard) venom, which causes local tissue necrosis and significant histological alterations [48]. Therefore, the toxic effects on muscle fibres and functional consequences induced by spider venoms may arise due to both catalytically active and/or non-enzymatic components. The absence of the most common myotoxic enzymes in *P. regalis* venom suggests the active role of small peptides in inducing myotoxicity, likely through the deregulation of ion channels. Moreover, we cannot rule out the possibility of the presence of other proteases, phospholipase isoforms, and non-enzymatic muscle-damaging components in this venom. It is also generally expected that invertebrate venoms are often abundant in small molecular weight compounds including antimicrobial peptides and cytosine-rich peptides [44]. Future studies on individual venom components are required to prove their effects on skeletal muscle.

Furthermore, *P. regalis* venom was unable to affect the viability and migration of C2C12 myoblasts, but it attenuated the fusion of these cells into myotubes and induced atrophy. Additionally, in the atrophy assays, which contain a mixture of myotubes and myoblasts, only the myotubes were affected by the venom. This suggests that undifferentiated myoblasts are not impacted by the venom, while differentiated cells are more susceptible. The use of the C2C12 cellular model for understanding venom myotoxicity has been widely reported for snake venoms more than for invertebrate species [49,50]. A few studies on scorpion venoms were reported using these cells to determine their effects on myoblasts and/or myotubes [51,52]. To the best of our knowledge, the comparison of the susceptibility of differentiated myotubes and muscle precursor cells to spider venoms has not previously been reported. Morphological changes together with changes in intracellular Ca^2+^ levels and biomarker release have been reported for invertebrate venom-induced muscle damage using in vitro experiments in murine muscle cell line [52,53]. On the other hand, for the effects of snake venoms, a larger number of venoms and their isolated toxins have been tested and compared at both stages of muscle development using in vitro and in vivo experimental approaches [19,51,54]. In line with our data, some previous findings suggest that differentiated myotubes are generally more affected by the toxic components of snake venoms than myoblasts [51,54]. The basis of this differential toxicity to myotubes and myoblasts is not yet fully understood, and therefore, further research is required to underpin these differential effects. However, one possibility to explain this differential response could be the appearance of molecular structures during the differentiation process, in particular those responsible for excitation–contraction coupling (ECC) [including the ryanodine (RyR) receptor and the dihydropyridine (DHPR) receptors] [55]. Previous studies have shown that invertebrate toxins such as maurocalcine, a 33-amino-acid peptide isolated from the venom of a scorpion, *Scorpio maurus palmatus,* can penetrate cell membranes and target the RyR of the ECC apparatus leading to leakage of calcium from intracellular stores [56,57]. These events may alter the calcium balance in the cytoplasm and possibly lead to the activation of calcium-sensitive intercellular proteases (calpains) and initiate cellular degradation [58]. Hence, the impact of *P. regalis* venom on intracellular calcium levels and signalling relevant to the differentiation of myoblasts into myotubes should be investigated in future research.

Under in vivo settings in mice, *P. regalis* venom was capable of inducing significant skeletal muscle damage, as demonstrated by around a 40% loss in muscle weight within 5 days following venom administration. Thereafter, the innate muscle regeneration underpinned by the resident muscle stem cells (satellite cells) was able to reconstitute the tissue to a near-normal condition within roughly three weeks. This was not unexpected given the muscles’ rapid regenerative capacity. Indeed, our previous work has demonstrated the ability of the TA muscle to fully recover from complete muscle damage with cardiotoxin within 10 days [19]. Furthermore, mature myofibres, which are more differentiated than myotubes, are necrotised by *P. regalis* venom in mice. One likely scenario to link these findings may be the dysregulation of intracellular Ca^2+^ ions and the maturation of cellular programmes in muscles that are sensitive to Ca^2+^. Skeletal muscle is dependent on a large number of physiological functions including contraction [59]. Skeletal muscle contains large Ca^2+^ stores, which must be carefully regulated. Dysregulation, for example changes in intracellular Ca^2+^ levels, resulted in sarcolemma damage and activation of Ca^2+^ sensitive proteases, which can degrade the cellular proteins leading to fibre necrosis [60]. The toxin 2 (PhTx2) isolated from the venom of a South American spider, *Phoneutria nigriventer* has been found to cause the hypercontraction of muscle fibres and high cytosolic Ca^2+^ levels in addition to clumped myofibrils with lost myofibrillar content pointing towards ruptured sarcolemma [61]. Therefore, the impact of *P. regalis* venom may become more pronounced during the development of muscle fibres as cells become more dependent on Ca^2+^ mediated processes that are accompanied by the generation of remedial programmes should its level become supraphysiological. Further studies to determine the impact of *P. regalis* venom on intracellular Ca^2+^ levels would reveal the direct actions of venom toxins in muscle damage due to their actions on Ca^2+^ levels.

The important regeneration stage of the clearance of necrotised fibres by the cells of the innate immune system is evidenced by IgG infiltration and is also supported by the presence of macrophages. However, there may be other mononucleated cells in the infiltrate, as shown by the DAPI staining surrounding regenerating fibres. This is suggestive of a mixed cellular response, likely from other cells of the immune system. Such other cells could include neutrophils, which rapidly arrive at an area of damage and assist in the recruitment of macrophages, while being involved in the clearance of necrotic tissue. However, the presence of neutrophils, perhaps due to venom components in the tissue can themselves be responsible for the damage and promotion of a proinflammatory environment [62]. Some earlier studies have evidenced the positive impact and therapeutic potential of intermittent regimens of glucocorticoids to treat muscular pathologies with similar inflammatory profiles of venom-induced muscle damage [63]. Therefore, investigations into whether anti-inflammatory drugs might facilitate myofiber repair by promoting a pro-regenerative response and reducing the time until regeneration is completed may be a direction for future studies.

Cardiotoxin (CTX) is a well-recognised venom-derived molecule that has been successfully employed in muscle research with significant contributions to the understanding of the mechanisms behind the muscle regeneration process and its modulation [64]. Previous work on CTX-mediated muscle damage and repair has shown that members of the three-finger toxins in snake venoms are key molecules that induce pore formation in the sarcolemma by binding to lipids and forming oligomers leading to the dysregulation of muscle function [65]. Nevertheless, some candidate pore-forming toxins, albeit not classical three-finger toxins, have been found in some spider species, and they are regarded as antimicrobial toxins due to their activity against bacteria [66]. Membrane-damaging peptides such as LaFr26, C1T1, and oxyopinin-2b in spider venoms act by membrane-disrupting mechanisms that lead to changes in the architecture of this biological barrier and its functions with subsequent cell death [67]. It has been proposed that these venom cationic peptides are used as chemical strategies to directly attack and destroy the lipid–membrane bilayer for defensive and digestive purposes [68]. A comprehensive biochemical profile of *P. regalis* venom is needed to establish the presence of specific molecules with pore-forming properties in this venom as these toxins may be responsible for this venom-induced muscle damage.

Our in vivo results indicate that *P. regalis* venom induces muscle damage and necrosis. We note that damage was induced in regions close to the site of venom injection, and more distal sites were unaffected. Therefore, there is a spatial as well as temporal aspect to *P. regalis* venom-induced muscle damage. The localised nature of the muscle damage could be related to either the lack of long-acting venom components or that the venom is rapidly cleared. We have previously shown that a metalloprotease from the venom of *Crotalus atrox* is capable of persisting in muscle for over 10 days, a property mediated most likely by its ability to bind to extracellular matrix components [19]. The long-acting venom was responsible for continual degeneration followed by regeneration, which eventually failed resulting in fibrogenesis. Furthermore, we have previously shown that repeated injection of CTX is capable of deregulating the otherwise robust regeneration programmes resulting in tissue fibrosis [69]. In this study, the muscle, on the whole, regenerates well at 20 days, and therefore, we propose that most of the highly potent molecules in the venom have a limited period of activity. Indirect effects of *P. regalis* venom on muscle include bleeding and the activation of platelets resulting in the formation of blood clots/microthrombi. While reduced from their peak area on day 5, the microthrombi are still present on day 20. This suggests that microthrombi may be a chronic feature of *P. regalis* envenomation. A previous study reported the coagulation-promoting properties of a recombinant peptide (LCTX-F2) derived from the venom of *Lycosa singoriensis,* and this was considered the first procoagulant peptide identified in spider venom. This bioactive molecule significantly reduces the activated partial thromboplastin time, potentiating the proteolytic activities of different blood coagulation factors, such as kallikrein, FXIIa, thrombin and FXa [70]. Thus, consideration must be given to the long-term effects of microthrombi induced by *P. regalis* venom and its modulatory effects on the enzymatic activities of blood coagulation factors in future studies.

## 5. Conclusions

In summary, this study demonstrates that *P. regalis* venom induces significant acute muscle damage and impedes muscle regeneration at early time points although it allows regeneration later. We have also identified the key stages of muscle regeneration that are impacted by this venom at the early time point. The venom shows no significant impact on the early processes of muscle fibre regeneration but impedes the fusion of myoblasts and induces atrophy. Further knowledge of the underlying mechanisms of the venom-induced effects through in vitro and in vivo assays could help develop suitable treatments for *P. regalis* envenomation. Overall, the biochemical, functional, and structural characterisation of understudied invertebrate venoms will not only open perspectives for a better understanding of the pathology and management of envenomation but also offers an opportunity to select molecular tools to dissect biological processes associated with muscle development and muscle-related disorders in the future. Hence, future studies are required to identify the muscle-damaging molecules in *P. regalis* venom and their potential clinical and biotechnological applications.

## Data Availability

All data from this study are included within this manuscript.

## Supplementary Materials

The following supporting information can be downloaded at: https://www.mdpi.com/article/10.3390/cells12162074/s1, Figure S1: Biochemical characterisation of *P. regalis* venom. Figure S2: Macrophage staining in venom-damaged muscle sections.
