# Correction: Richards et al. Indian Ornamental Tarantula (*Poecilotheria regalis*) Venom Affects Myoblast Function and Causes Skeletal Muscle Damage. *Cells* 2023, *12*, 2074

**DOI:** 10.3390/cells14030191

**Published:** 2025-01-27

**Authors:** Nicholas J. Richards, Ali Alqallaf, Robert D. Mitchell, Andrew Parnell, Husain Bin Haidar, José R. Almeida, Jarred Williams, Pradeep Vijayakumar, Adedoyin Balogun, Antonios Matsakas, Steven A. Trim, Ketan Patel, Sakthivel Vaiyapuri

**Affiliations:** 1 School of Biological Sciences, University of Reading, Reading RG6 6UB, UK; n.j.richards@pgr.reading.ac.uk (N.J.R.); a.alqallaf@pgr.reading.ac.uk (A.A.); andrewparnell@micregen.com (A.P.); h.m.binhaidar@pgr.reading.ac.uk (H.B.H.); 2 Medical Services Authority, Ministry of Defence, Kuwait City 13012, Kuwait; 3 Micregen Ltd., Thames Valley Science Park, Reading RG2 9LH, UK; robertmitchell@micregen.com; 4 School of Pharmacy, University of Reading, Reading RG6 6UB, UK; j.r.dealmeida@reading.ac.uk (J.R.A.); j.williams4@pgr.reading.ac.uk (J.W.); pradeep.vijayakumar@pgr.reading.ac.uk (P.V.); 5 Molecular Physiology Laboratory, Centre for Biomedicine, Hull York Medical School, Hull HU6 7RX, UK; adedoyin.balogun@nhs.net (A.B.); antonios.matsakas@hyms.ac.uk (A.M.); 6 Venomtech Ltd., Sandwich CT13 9FE, UK; s.trim@venomtech.co.uk

## Error in Figure

In the original publication [1], there was a mistake in Figure 1 as published. In Figure 1C, the panels for “4 hours” and “24 hours” were the same. The corrected Figure 1 appears below. The authors state that the scientific conclusions are unaffected. This correction was approved by the Academic Editor. The original publication has also been updated.

## Figures and Tables

**Figure 1:**
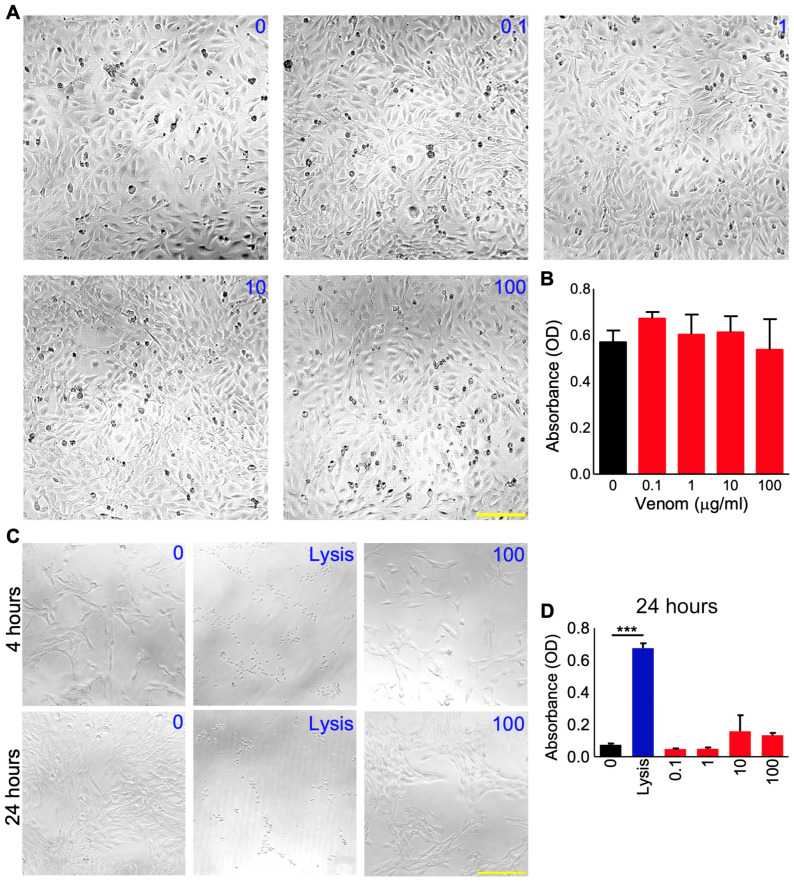
Effects of *P. regalis* venom on the survival of C2C12 myoblast cells. The morphology (**A**) and viability (assessed using an MTS reagent) (**B**) of C2C12 cells were analysed after 24 h of incubation with various concentrations (0 to 100 µg/mL) of venom. Similarly, (**C**) the effects of a range of concentrations of venom on C2C12 cells were assessed using a lactate dehydrogenase assay at four and 24 h following treatment. A lysis buffer containing Triton-X 100 (Lysis) was used as a positive control in this assay. The images shown are representative of three independent experiments, and the quantified data (**D**) show the level of absorbance obtained in this assay. Data represent the mean ± SEM (*n* = 3). The scale bars represent 200 µm and are applicable to all the images in each panel. The *p*-value shown (*** *p* < 0.001) was calculated using one-way ANOVA.
