# Supplementary material for: Indian Ornamental Tarantula (Poecilotheria regalis) Venom Affects Myoblast Function and Causes Skeletal Muscle Damage
Source: Cells. 2023 Aug 15;12(16):2074. doi: 10.3390/cells12162074 (PMC10453882; doi:10.3390/cells12162074)
Supplement: Supplementary file 1 [file cells-12-02074-s001.zip › cells-2473978-SI.pdf]

Supplementary information

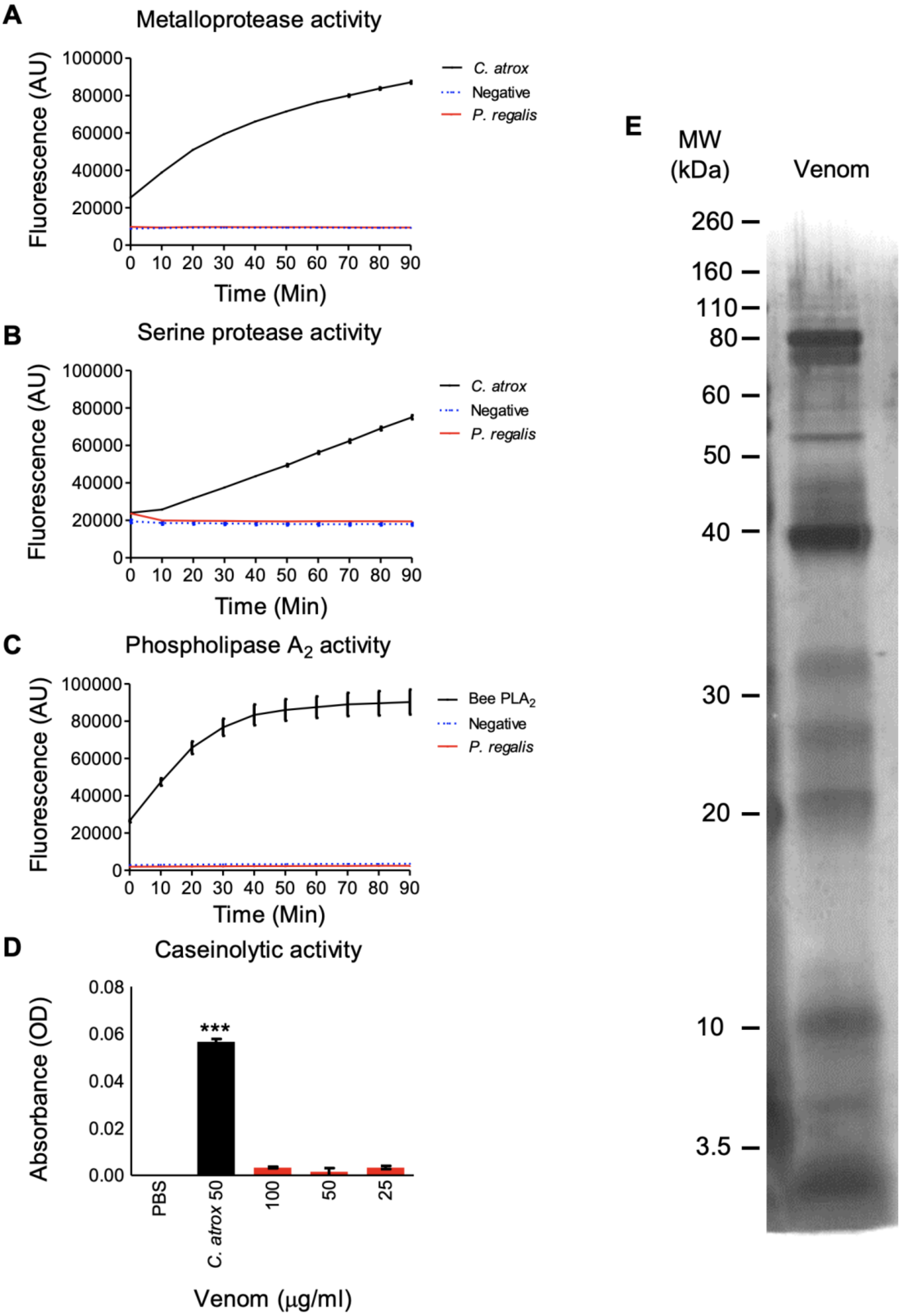

Figure S1. Biochemical characterisation of *P. regalis* venom. (**A**) Metalloprotease and (**B**) serine protease activities of 50 µg/ml *P. regalis* venom compared to *Crotalus atrox* snake venom as a positive control were analysed using DQ gelatin and BAAMC as substrates, respectively by spectrofluorimetry. **C)** Phospholipase A_2 activity of 50 µg/ml *P. regalis* venom compared to bee venom as a positive control was measured using an EnzCheck PLA_2 assay kit by spectrofluorimetry. The activities were measured at 10-minute intervals for 90 minutes. **D)** Caseinolytic activity of 100, 50 and 25 µg/ml of *P. regalis* venom compared to *Crotalus atrox* snake venom as a positive control was measured using azocasein and the absorbance was measured at 90 minutes following incubation by spectrofluorimetry. Data represent mean ± SEM (n=4). The *p* value shown was calculated by one-way ANOVA followed by Bonferroni post-test. **E**) Whole *P. regalis* venom (10 µg) was subjected to SDS-PAGE followed by silver staining. Novex® Sharp Pre-Stained Protein Standard was used as a molecular weight (MW) marker.

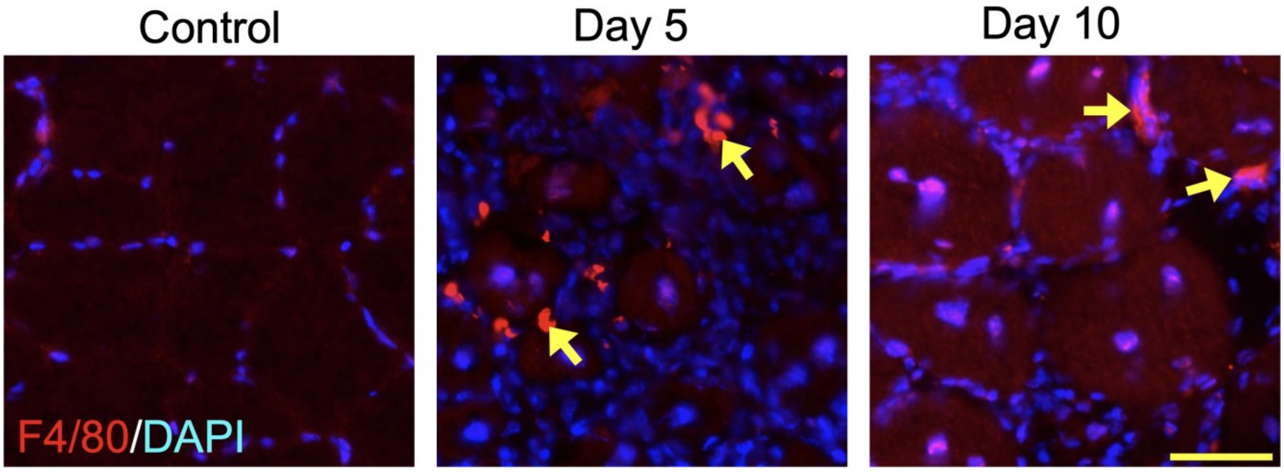

Figure S2. F4/80 staining for macrophages in venom-damaged muscle sections. F4/80 staining to visualise macrophages in control and venom-damaged TA muscles at days 5 and 10 was performed using selective antibodies. The images shown are representative of four independent experiments using muscle sections from different mice. The scale bar represents 50 μm.
